# Supplementary material for: Designing integrated care models for mental health and tuberculosis in Pune, India: A formative qualitative study of patient, caregiver and provider perspectives
Source: Glob Ment Health (Camb). 2026 Jan 2;13:e7. doi: 10.1017/gmh.2025.10123 (PMC12835940; doi:10.1017/gmh.2025.10123)
Supplement: Poddar et al. supplementary material [file S2054425125101234sup001.docx]

**ANNEXURE 1: Codebook used for data analysis with coding description and frequency**

| **Name** | **Description** | **Files** | **References** |
| --- | --- | --- | --- |
| INDI (Individual) | | 0 | 0 |
| Innovation deliverers | Individuals who are directly or indirectly delivering the innovation. | 14 | 36 |
| Knowledge and Beliefs | Individuals’ attitudes toward and value placed on the integrated services, as well as familiarity with facts, truths, and principles related to the integrated services. | 16 | 25 |
| Self-efficacy | Individual belief in their own capabilities to execute courses of action to achieve implementation goals, as they related to service design, co-creation or implementation. | 11 | 17 |
| INNER (Inner setting) | | 0 | 0 |
| Available resources | The level of resources organizational dedicated for implementation and on-going operations including physical space and time. | 13 | 29 |
| Compatibility | The degree of tangible fit between meaning and values attached to the proposed integrated services by involved individuals, how those align with individuals’ own norms, values, and perceived risks and needs, and how the services fit with existing workflows and systems. | 2 | 2 |
| Culture | Norms, values, and basic assumptions of a given Tuberculosis Unit or health center. | 3 | 4 |
| Implementation Climate | The absorptive capacity for change, shared receptivity of involved individuals to the proposed integrated services, and the extent to which use of that innovation will be rewarded, supported, and expected within the tuberculosis unit or health center. | 1 | 2 |
| Network and Communication | The nature and quality of webs of social networks, and the nature and quality of formal and informal communications within a Tuberculosis Unit or health center. | 2 | 2 |
| Organizational incentives and rewards | Extrinsic incentives such as goal-sharing, awards, performance reviews, promotions, and raises in salary, and less tangible incentives such as increased stature or respect. | 2 | 2 |
| Readiness for implementation | Tangible and immediate indicators of the tuberculosis unit or health center's commitment to its decision to implement the proposed integrated services. | 1 | 2 |
| INNO (Innovation) | | 0 | 0 |
| Adaptability | The degree to which the integrated services can be adapted, tailored, refined, or reinvented to meet local needs. | 3 | 4 |
| Complexity | Perceived difficulty of the integrated services, reflected by duration, scope, radicalness, disruptiveness, centrality, and intricacy and number of steps required to implement. | 22 | 66 |
| Cost | Costs of the integrated services and costs associated with implementing the integrated services including investment, supply, and opportunity costs. | 5 | 6 |
| Relative Advantage | Stakeholders’ perception of the advantage of implementing the integrated services versus an alternative solution. | 23 | 47 |
| Sustainability | The likelihood of continued use of integrated intervention components and activities for the continued achievement of desirable outcomes. | 2 | 3 |
| INTE (Integration)  Proposed interventions or ideas that aim to integrate MH services with TB care | | 7 | 8 |
| Counselling |  | 18 | 44 |
| Education and Awareness |  | 19 | 65 |
| Group Sessions |  | 7 | 10 |
| Home visits |  | 6 | 7 |
| Medication |  | 6 | 6 |
| MH Screening |  | 8 | 12 |
| Monitoring |  | 1 | 1 |
| Referrals |  | 8 | 11 |
| Sharing experiences |  | 18 | 32 |
| MH PER (Mental health perceptions) | | 16 | 43 |
| OUTER (Outer setting) | | 0 | 0 |
| Community Characteristics | The extent to which community characteristics affect the willingness or ability for tuberculosis units or health centers to engage in implementation. | 2 | 2 |
| External Policy and Incentives | A broad construct that includes external strategies to spread innovations including policy and regulations (governmental or other central entity), external mandates, recommendations and guidelines, pay-for-performance, collaboratives, and public or benchmark reporting. | 11 | 21 |
| Local Attitudes | Attitudes, values of beliefs in the local community about MH or TB that can influence implementation. (Example: stigma related to TB or MH conditions). | 19 | 39 |
| Patient needs and resources |  | 5 | 10 |
| PATH (Pathways)  Patient journey to seeking care, treatment, post-treatment pathways | | 0 | 0 |
| Diagnosis |  | 15 | 21 |
| MH experiences |  | 15 | 49 |
| Post-treatment |  | 3 | 7 |
| Pre-treatment evaluation |  | 4 | 5 |
| Treatment |  | 20 | 44 |
| PROCESS (Process) | | 1 | 1 |
| Decision Making | The type, duration and timing of the activities involved in making decisions about the proposed integration service. | 4 | 6 |
| Engaging | Attracting or involving appropriate individuals or stakeholders in the implementation and use of the intervention through social marketing, education, role modeling, training, and other similar activities. | 19 | 53 |
| Executing | Carrying out or accomplishing the implementation according to plan. | 4 | 7 |
| Innovation Participants | Individuals served by the organization that participate in the integrated service, e.g., patients in a prevention program in a hospital or TB survivors. | 15 | 20 |
| Key Stakeholders | Individuals from within the tuberculosis unit or health center that are directly impacted by the proposed integrated service, e.g., staff responsible for making referrals to a new program or using a new work process. | 6 | 6 |

*Note: Deductive (CFIR) codes are outlined in blue and inductive codes are outlined in yellow*
